# COVID-19 Vaccines: “Warp Speed” Needs Mind Melds, Not Warped Minds

**DOI:** 10.1128/JVI.01083-20

**Published:** 2020-08-17

**Authors:** John P. Moore, P. J. Klasse

**Affiliations:** a Department of Microbiology and Immunology, Weill Cornell Medical College, New York, New York, USA; Emory University

**Keywords:** SARS-CoV-2, S-protein, RBD, COVID-19, neutralizing antibodies, serology, vaccines, animal models, Warp Speed

## Abstract

In this review, we address issues that relate to the rapid “Warp Speed” development of vaccines to counter the COVID-19 pandemic. We review the antibody response that is triggered by severe acute respiratory syndrome coronavirus 2 (SARS-CoV-2) infection of humans and how it may inform vaccine research. The isolation and properties of neutralizing monoclonal antibodies from COVID-19 patients provide additional information on what vaccines should try to elicit. The nature and longevity of the antibody response to coronaviruses are relevant to the potency and duration of vaccine-induced immunity. We summarize the immunogenicity of leading vaccine candidates tested to date in animals and humans and discuss the outcome and interpretation of virus challenge experiments in animals. By far the most immunogenic vaccine candidates for antibody responses are recombinant proteins, which were not included in the initial wave of Warp Speed immunogens. A substantial concern for SARS-CoV-2 vaccines is adverse events, which we review by considering what was seen in studies of SARS-CoV-1 and Middle East respiratory syndrome coronavirus (MERS-CoV) vaccines. We conclude by outlining the possible outcomes of the Warp Speed vaccine program, which range from the hoped-for rapid success to a catastrophic adverse influence on vaccine uptake generally.

## Abstract (precis)

In this review, we address issues that relate to the rapid “Warp Speed” development of vaccines to counter the COVID-19 pandemic. We review the antibody response that is triggered by severe acute respiratory syndrome coronavirus 2 (SARS-CoV-2) infection of humans and how it may inform vaccine research. The isolation and properties of neutralizing monoclonal antibodies from COVID-19 patients provide additional information on what vaccines should try to elicit. The nature and longevity of the antibody response to coronaviruses are relevant to the potency and duration of vaccine-induced immunity.

## INTRODUCTION

An effective vaccine is the best long-term solution to the COVID-19 pandemic. Worldwide, governments are responding by investing in the research, testing, production, and distribution programs required to make a vaccine and are doing so with highly aggressive timelines (https://www.who.int/who-documents-detail/draft-landscape-of-covid-19-candidate-vaccines). In the United States, the words “Warp Speed” were adopted by politicians to indicate the urgency of the need for a vaccine. If a vaccine is proven effective even as early as the first few months of 2021, then the accomplishment will have sliced many years off the usual timeline for vaccine development. The need for speed is understandable, but it comes with risks: specifically, the vaccine candidates that are most capable of rapid production on the required massive scale may not be the most effective, and there are concerns that immune responses to COVID-19 vaccines could enhance infection or exacerbate disease in individuals who become infected despite vaccination. These topics have been raised in multiple perspectives and reviews in recent weeks (1–12). Here, we attempt to address key subjects in greater detail, with an emphasis on quantitative aspects of antibody-based immune responses to severe acute respiratory syndrome coronavirus 2 (SARS-CoV-2). The literature on COVID-19 expands daily, so a review like this is out-of-date the moment both fingers cease tapping the keyboard. We have relied not just on peer-reviewed publications but also on manuscripts deposited on preprint servers, in the full knowledge that some information in some of those reports may be inaccurate. Accordingly, we urge readers to inspect key papers themselves, particularly in their final peer-reviewed forms. We also recommend using an additional resource for judging some preprints (13). We regret that we have, no doubt, overlooked key papers among the daily torrent.

**FIG 1:**
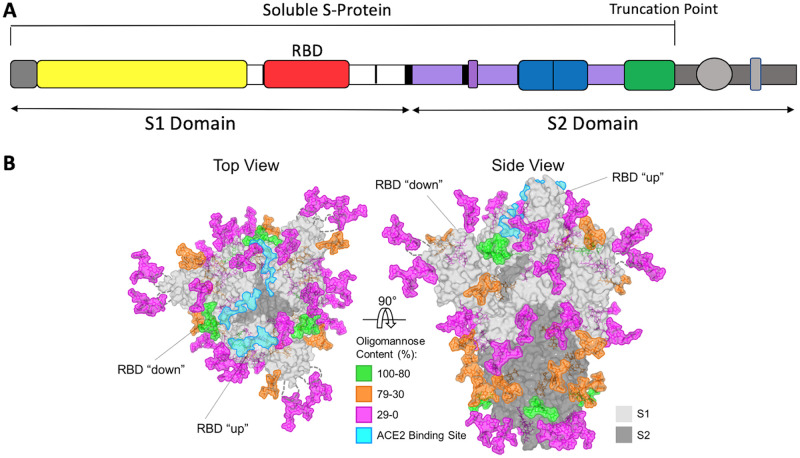
The SARS-CoV-2 S-protein. (A) Schematic of the S-protein showing the S1 and S2 domains and the RBD. The soluble S-protein ends at the engineered truncation point. The areas colored in gray indicate the transmembrane and intracytoplasmic domains that are present in the full-length S-protein on virions. The most commonly used immunogens are the soluble S-protein, the S1 domain, and the RBD, although some nucleic acid and viral vector constructs are based on the full-length S-protein. (B) Structure-based representation of the S-protein trimer viewed from above and the side, as indicated. The protein surface is in gray, with the ACE2 binding site on the RBD highlighted in aquamarine. On one protomer, the RBD is shown in the “up” position, while on the other two it is in the “down” position, as indicated. Glycans are colored according to the scale, based on their oligomannose content. Adapted from reference 77 under a CC BY 4.0 license.

Most of the COVID-19 vaccines in development are intended to induce antibody responses that neutralize SARS-CoV-2, thereby preventing it from entering target cells and infecting the host. In some cases, the vaccines may also induce antibody and/or cellular immune responses that can kill and eliminate already infected cells, thereby limiting the replication of the virus within a transiently infected host. Nonetheless, most emphasis is being placed on the induction of virus-neutralizing antibodies (NAbs) directed against the SARS-CoV-2 spike (S) protein (Fig. 1). The immunogens used to elicit NAbs are various forms of the S-protein, including the isolated receptor-binding domain (RBD) (14–18). The S-proteins can be expressed *in vivo* from DNA or mRNA constructs or by recombinant virus vectors such as adenovirus or vaccinia. Alternatively, they can be directly delivered as recombinant proteins with or without an adjuvant or as a constituent of a killed virus vaccine (Table 1). All of these methods, and more, are included in the hundreds of vaccine programs now at the preclinical and animal model stages, and they are represented among the now very-high-profile programs being ramped up in different countries (https://www.who.int/who-documents-detail/draft-landscape-of-covid-19-candidate-vaccines). Some nations are working together in consortia, although the United States appears to be adopting a go-it-alone policy (19). The American “Warp Speed” program was reported to have been narrowed down to five front-line candidates that are based on mRNA (Moderna, Pfizer) or adenovirus and other viral vectors (Oxford University/AstraZeneca, Janssen, and Merck) (20). However, decisions in this area change rapidly, and a U.S. government website should be consulted for updated information (https://medicalcountermeasures.gov/app/barda/coronavirus/COVID19.aspx). One S-protein-based vaccine, made in insect cells by Protein Sciences/Sanofi, is listed.

**TABLE 1:** Categories of vaccines for protection against SARS-CoV-2 infection and/or disease^*a*^

Vaccine category	Safety^*b*^	Speed and ease of production	Logistics of global distribution	Potential for NAb induction	Potential for cell-mediated immunity^*c*^
Live attenuated virus	Substantial concerns	NA^*d*^	NA	Probably high	Probably good
Inactivated virus	Some concerns^*e*^	Intermediate	Feasible	Moderate	Poor
Nonreplicating virus vector (recombinant DNA virus)	High	High	Feasible	Weak	Probably good
DNA plasmid given by electroporation	High	High	Some concerns^*f*^	Very weak	Probably good
mRNA	High	High	May be difficult^*g*^	Weak	Probably good
Soluble or nanoparticle S- or RBD-protein, with adjuvant	High	Low^*h*^	Feasible	High	Poor

a For a complete list of vaccine candidates in preclinical and phase 1/2/2b/3 clinical trials, see https://www.who.int/publications/m/item/draft-landscape-of-covid-19-candidate-vaccines. All the categories listed in the table are represented except live attenuated virus, which is a traditional and widely used method that is not being tested for SARS-CoV-2. How the various categories are summarized in this table is based on the small amount of available data, combined with general experience of how similarly designed vaccines have performed against other viral pathogens. Nonetheless, there are considerable uncertainties behind some of the assessments in the table. Emerging clinical trial data will determine whether they are accurate.

b Safety indicates the likelihood the vaccine will be tolerated without serious adverse effects in the absence of infection. For all categories, there are substantial uncertainties about the risk of exacerbated pathogenesis postinfection, by ADE and VAERD mechanisms (see the text). These risks may be the greatest for vaccines that induce only low NAb titers and/or a high non-NAb/NAb ratio.

c Most emphasis has been placed on the induction of NAbs, although some data on cellular immune responses are emerging from animal studies and more will be obtained in human trials. Attempts to induce cytotoxic T cells might include immunization with viral proteins other than S, including nonsurface exposed internal ones (e.g., the N-protein). Extrapolation from other vaccines leads to the assessments listed.

d NA, not applicable. There are no known plans to produce this type of vaccine.

e For a killed virus vaccine to be safe, the pathogen must be fully inactivated. Historically, inactivation has sometimes been incomplete (e.g., with polio vaccines).

f Delivering DNA vaccines into muscles via electroporation is a relatively complex procedure compared to direct injection via needles or oral delivery.

g The ease with which mRNA vaccines can be formulated and distributed has not been widely discussed. However, if these vaccines turn out to be unstable at ambient temperatures, it will be challenging to distribute frozen or chilled stocks.

h General experience suggests that producing a stable cell line and using it to make large stocks of recombinant proteins under good manufacturing process conditions can take 1 to 2 years.

**FIG 2:**
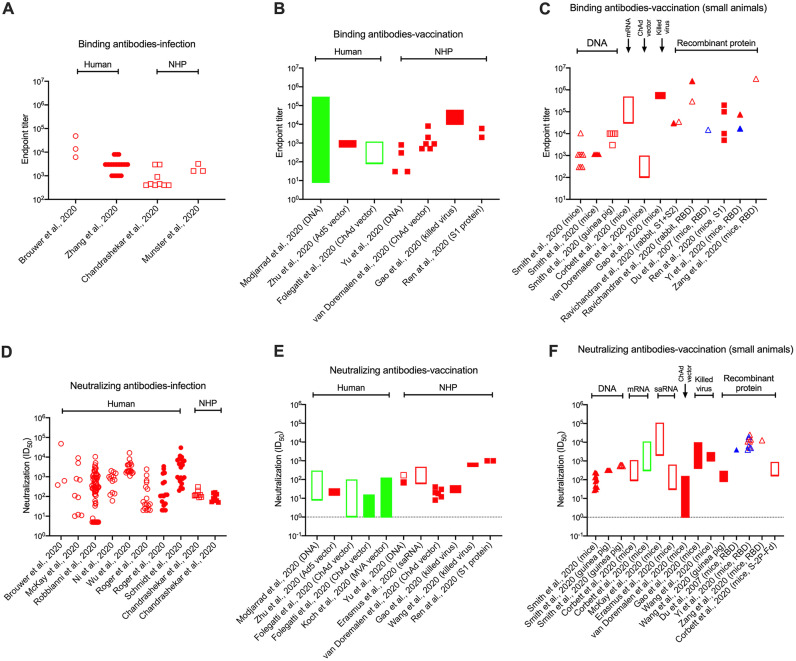
Magnitudes of S-protein binding antibody (ELISA) and NAb responses in COVID-19 cases and vaccinated humans and animals. (A to C) Anti-S protein (open symbols) and anti-RBD (closed symbols) endpoint titers. (D to F) NAb midpoint titers (ID_50_) from PV assays (open symbols) and RV assays (closed symbols). In each plot, the titers for individual study subjects, the median values for a test group, or the range recorded in a study cohort are presented. The data in panels A and D are derived from virus-infected humans and nonhuman primates (NHPs) and show titers obtained in the first several weeks post symptoms. (B, C, E and F) Peak responses to S-protein- or RBD-based vaccines in humans and animals. (B and E) Studies in humans and NHPs; (C and F) studies in small animals (mice, guinea pigs, and rabbits), as indicated by the labels on the *x* axes. In the small-animal experiments, the immunogens used are grouped together from left to right as follows: DNA, RNA, adenovirus vectors, killed virus, recombinant S-protein, or RBD-protein. Data relating to SARS-CoV-2 are in red, SARS-CoV-1 in blue, and MERS-CoV in green. For experimental details, the cited papers listed on the *x* axes should be consulted. Assay methodologies vary between studies, which reduces the comparability of the resulting data sets. However, we judge that broad trends can still be seen. We have only included binding antibody endpoint titers and NAb midpoint (ID_50_) titers on the plots, excluding other methods of data representation. Multiple other papers cited in the text report on antibody responses to the S-protein (or other antigens) in infected humans but do so using other formats; in those papers, the responses usually span a >1,000-fold range. We note that NAb endpoint titers were presented in the following papers and the unrecorded midpoint titer values would probably be >100-fold lower: endpoint titer range <10 to ∼300 for MERS-CoV DNA vaccine-immunized humans (103); median endpoint titers of 34 and 46 in RV and PV assays, respectively, for Ad5 vaccine-immunized humans (94); endpoint titer range of 5 to 60 for SARS-CoV-2-infected rhesus macaques (128); median endpoint titer of ∼40 for ChAdOx1-immunized rhesus macaques (131).

Both binding antibody (enzyme-linked immunosorbent assay [ELISA]) and NAb responses are quantified and presented in different ways in different studies of SARS-CoV-2 infection or vaccination. The most useful method involves deriving endpoint or midpoint titers from titration curves, but that is not always done and alternative measurements are quite common. Moreover, it is often not specified whether a titer is an endpoint or a midpoint, which matters greatly when trying to judge the relative immunogenicity of different vaccine candidates (see below) (Fig. 2). Endpoint titers can be orders of magnitude higher than midpoint (half-maximal inhibitory dilution-factor [ID_50_]) values, depending on which cutoff is chosen and the slope of the curve. NAb responses to vaccines are presented in some papers as endpoint titers (higher numbers), which should be born in mind in comparisons with other vaccines for which NAb data are reported as midpoint titers (much lower numbers). Variation in how laboratories generate ELISA and NAb data further complicates cross-study comparisons. When possible, we specify whether a titer value is an endpoint or a midpoint or make an educated guess. Titers are not always recorded in the text of papers; in those cases, we have estimated key values by visual inspection of plotted data. It is to be hoped that standardized methods of data generation and presentation will be used in the Warp Speed and other national vaccine development programs.

## ANTIBODY RESPONSES IN COVID-19 CASES VARY GREATLY DURING THE CLINICAL COURSE

Nearly all SARS-CoV-2-infected people develop IgM, IgG, and IgA antibodies against the viral nucleocapsid (N)- and S-proteins between 1 and 2 weeks post symptoms; the titers of antibodies, including sometimes NAbs, then remain elevated for at least several weeks after the virus is no longer detectable and the patient recovers (15, 17, 21–23). Titer decay rates over long periods have yet to be reported (see below). In one study, all 20 convalescent patients had virus-specific CD4^+^ T cells and, in 70%, a measurable CD8^+^ T-cell response. The magnitude of the S-protein-specific CD4^+^ T-cell response in that cohort correlated with IgG and IgA titers against the RBD, suggesting that the antibody response to SARS-CoV-2 is, as expected, T-help dependent (24). Endpoint titers of anti-S protein IgG Abs are highly variable in acute and convalescent COVID-19 cases, ranging from undetectable to >100,000 (25–31).

Both Env-pseudotype virus (PV) and, less often, replicating virus (RV) assays are used for NAb quantitation. Studies that compare these two formats generally show concordance with respect to rank orders for test antibodies, with the PV assays usually but not always being a fewfold more sensitive (25, 32–35). NAb titers are best reported as ID_50_ values and also vary greatly for COVID-19 sera. Midpoint (ID_50_) titers in COVID-19 sera span the range from undetectable to >10,000, although titers of >5,000 are uncommon (25, 27, 33, 34, 36, 38). In one study of 22 convalescent COVID-19 patients, NAb midpoint titers ranged from below detection (<30) to 1,900 (33). The median NAb titer was ∼1,000 in a larger cohort of 175 patients who had recovered from mildly symptomatic COVID-19; in only 10 cases were NAbs undetectable, while 25 had midpoint titers of >2,500 (38). The highest recorded titers in three studies were ∼1,000 (30), 21,000 (38), and ∼3,000 (36). Similar variation was found in another cohort study in which the extent of neutralization was measured in a PV assay (39). In general, measurements of anti-S and anti-RBD IgG antibodies, but sometimes also antibodies to the neutralization-irrelevant N-protein, correlate quite well with the output of neutralization assays, although sometimes the analyses are not titer-to-titer comparisons (26, 27, 33, 38, 39, 41). Thus, neutralization-relevant epitopes on the RBD are highly antigenic and immunogenic, but so are other epitopes elsewhere on the S-protein that are less associated with neutralization (25–27, 29, 43, 53). One reason is that some epitopes may be antigenic on recombinant S-proteins but not on the functional virion-associated spike. Taken together, the anti-S protein antibody and NAb measurements in COVID-19 cohorts are a useful frame of reference for interpreting vaccine trials in animals and humans (see below). Quantitative aspects of the infection- and vaccine-induced antibody responses to S-proteins are summarized in Fig. 2.

The extreme variation in antibody titers seen in COVID-19 cases may reflect the pathological consequences of infection, which could limit the development of the antibody response. In some early studies, the samples may sometimes have been collected too early in the disease course, before the titers had reached their peak. Nonetheless, the titer variation holds true across multiple cohort studies of ever-increasing size and sophistication. What is also seen consistently is the lack of correlation between strong antibody responses and the amelioration of disease; indeed, the converse is true in that the highest antibody titers are seen in the patients who later develop the most severe disease and also in the oldest ones. In contrast, people with mildly symptomatic infection that did not require hospitalization generally have far weaker antibody responses (15, 21, 26, 38, 44). The same was seen in the SARS epidemic, where cases with the earliest and strongest NAb responses also had the poorest prognosis (45). A particularly striking example is a COVID-19 cohort study in which serum IgM, IgG, and IgA responses were stratified by disease status (22). Median anti-RBD antibodies, estimated as concentrations at their peaks, were IgA, 8.8 μg/ml on days 16 to 20; IgM, 7.2 μg/ml on days 16 to 20; and IgG, 16 μg/ml on days 21 to 25. Of note is that serum IgA concentrations were very strongly correlated with severe disease (*P* < 0.0001), much more so than for IgG (*P* < 0.001 for moderate disease but not significant for severe) and IgM (not significant). High serum IgA levels and their correlation with severe disease were also seen in three more COVID-19 cohorts (22, 23, 46). In one study, virus-specific IgA antibodies to the S-, N-, and RBD-proteins were detected a few days sooner than either IgM and IgG and initially dominated over these isotypes at the B-cell and serum antibody levels as infection progressed. Eventually, however, IgG titers rose to match IgA and then became the strongest response over the longer term (1 to 2 months). Unexpectedly, serum antibody fractionation experiments showed that both IgG and IgA isotypes had neutralization activity, with IgA being significantly stronger during peak infection. Bronchoalveolar lavage (BAL) samples also contained NAbs as well as IgA and IgG anti-S antibodies (46). These observations of high-titer serum IgA responses may be yet another novel aspect of SARS-CoV-2 infection. In contrast, infection by influenza viruses is not associated with unusually strong serum IgA responses (47).

NAb titers in the early stages of infection are inversely correlated with subsequent viral loads, measured as RNA copies in sputum, throat swabs, and stool, but directly correlated with more severe subsequent disease (48, 49). The relationships between the amount of infectious virus in key body compartments and disease severity and the influence of NAbs remain to be understood. It is worth noting, however, that infectious SARS-CoV-2 is very rarely found in blood, which is the body fluid used in most assessments of the antibody response to this virus. Conversely, antibody responses are only rarely measured in mucosal fluids, where infectious virus titers are far higher (49, 50). The same dichotomy generally applies in animal and human vaccine studies, where there is very little information on the induction of mucosal antibodies.

It is not known why earlier and stronger serum antibody responses correlate with disease severity (15). Do the higher viral loads and hence antigen supply associated with more severe disease drive antibody production, or do stronger antibody responses help drive the disease process (44, 51)? Although it is not clear what role antibodies play during SARS-CoV-2 infection, it is certainly not possible to identify what, if any, titers protect against disease in COVID-19 cohorts. This point is relevant because S-protein immunization studies in animals and humans often compare the magnitude of the anti-S binding antibody and NAb responses with what is seen in SARS-CoV-2-infected humans of sometimes unspecified disease status. Given the wide range of titers seen in infected people and how the strongest antibody titers are seen in the sickest patients, this kind of comparison is meaningless and not helpful (Fig. 2). Furthermore, the concept of “immunity passports” for infected people is often discussed. Only a minority of infected people develop severe symptoms; most recover fully at home. But, as noted above, minimally or moderately symptomatic individuals have far weaker antibody responses than hospitalized COVID-19 cases. It is very far from certain that these weak responses would be sufficient to protect against a second exposure, and for long (see below). Whether plasma from moderately symptomatic people could be useful for passive immunotherapy also needs to be considered, in view of the generally low NAb titers generated by such individuals.

## LONGEVITY AND PROTECTIVE CAPACITY OF ANTIBODY RESPONSES TO CORONAVIRUSES

**FIG 3:**
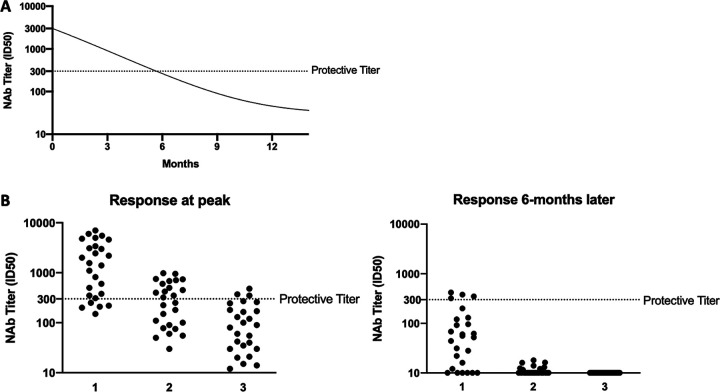
SARS-CoV-2 vaccine responses and their relationships to protective immunity. (A) The rate of decay of SARS-CoV-2 NAbs from an initial vaccine-induced peak ID_50_ titer of 3,000 during the following year. The titer intersects the protective titer value of 300 (dotted line) after 6 months. The values chosen are hypothetical, although a titer decay to below protective levels over a 6-month period would be broadly consistent with the decline after infection with common-cold coronaviruses (65–67). (B) Variation in SARS-CoV-2 NAb titers among a cohort of vaccinated individuals and the relationship to the protective titer value of 300 (dotted line). The value of 300 is hypothetical but is consistent with values discussed in the text (e.g., see reference 62). The assay has a titer quantitation limit of 10. (Left) Peak titers immediately after the immunization schedule is completed; (right) titers 6 months later. Scenario 1, the vaccine induces a strong enough peak response for most recipients to be protected and vaccine efficacy is high; scenario 2, for a weaker vaccine, the protective threshold is initially exceeded in only half of the recipients; scenario 3, titers in only a minority of the recipients of a poorly immunogenic vaccine exceed the protective threshold. In each scenario, the 50-fold titer decay over 6 months causes far fewer of the vaccine recipients to be protected at this time. A booster immunization for the two stronger vaccines could restore immunity to protective levels in some people. As for panel A, the titer values and decay rates are hypothetical. However, the range of titers seen in an immunization cohort is consistent with published data (103, 133, 134); an ∼50-fold decrease in the SARS-CoV-1 NAb titer during a 6-month period was measured in RBD-immunized mice (62), and NAb titers induced by a MERS-CoV DNA vaccine in humans had declined by ∼50-fold within a year (103).

A key question that has societal implications beyond vaccine development is whether the antibody response to SARS-CoV-2 will confer immunity against reinfection and, if so, for how long? Will humans who recover from this infection be protected against a future exposure to the same virus months or years later? Knowing the duration of the antibody response to SARS-CoV-2 vaccines will also help to determine whether, and how often, boosting immunizations will be needed if the initial response exceeds the protection threshold (see Fig. 3).

Antibody responses to many viral infections wane so slowly that lifelong immunity is maintained, with plasma cells and B-memory cells playing central roles in resistance to reinfection (17, 52). RBD-specific memory B cells that have switched to IgG were found in the blood of COVID-19 patients (17, 53). SARS CoV-2-specific plasma cells were identified in both severely ill patients and recent convalescent cases (17, 54). The SARS CoV-2 antibody responses in 47 patients were unchanged 2 weeks after their discharge from hospital (38). IgG antibodies to the S-protein were detected in all 31 COVID-19 patients soon after infection, rose during the first 3 weeks after symptom onset, and then declined but remained detectable at 8 weeks (55). Of necessity, the early studies on COVID-19 cohorts were conducted only over fairly short time periods. Longer-term assessments of antibody decay kinetics are now starting to emerge. In one cohort, antibody and NAb responses peaked, on average, at approximately days 30 to 40 postinfection and then began to decline over the next few weeks. In one individual, the highest NAb titer was ∼1,600 on day 20 but had fallen to ∼50 by day 45, which is a worrying rate of decay if seen more generally (56). In another report, IgA titers to the RBD-, S-, and N-proteins rose strongly for about 3 weeks postinfection and then rapidly declined to the extent that they were undetectable by 1 month postrecovery. In contrast, IgG titers were much more persistent in this time frame (46). Antibody responses (virus-specific IgG and NAbs in a PV assay) declined markedly in both symptomatic and asymptomatic individuals within 8 weeks after discharge from the hospital, to the extent that 13% of the former group and 40% of the latter become seronegative for virus-specific IgG in the assay used (21).

Until data on the persistence of antibody and NAb responses to SARS-CoV-2 over a multimonth period become available, we can only extrapolate from studies of SARS-CoV-1, Middle East respiratory syndrome coronavirus (MERS-CoV) and the common-cold coronaviruses (57). Antibody responses to SARS-CoV-1 dropped continuously during the first few years after infection (17, 57–60). In one study, IgG levels started to decline ∼6 months post symptoms and then fell steadily over the next 3 years (60). Virus-specific IgG remained detectable at a low serum dilution of one-tenth throughout a 13-year study of 34 SARS-CoV-1-infected health care workers but dropped to very low levels over this period and eventually approached the assay detection limit (54). S-protein-specific IgG memory cells were found at 2, 4, 6, and 8 months after SARS-CoV-1 infection, but their abundance fell by 90% between the first and last time points (61). Immunization of mice with the SARS-CoV-1 RBD protein induced very strong peak anti-S protein endpoint titers of ∼150,000 and NAb ID_50_ titers of 4,000. However, these titers declined to ∼10 and <40 within 9 months (62). Thus, well within a year, the antibody response to the RBD had dropped by as much as 15,000-fold.

Animal model experiments have started to address the nature of protective immunity to SARS-CoV-2. Two rhesus macaques experimentally infected on day 0 were protected from a second challenge on day 28, soon after they had recovered from their initial mild disease (63). A follow-up study on a larger scale (9 animals in 3 groups of 3) drew similar but more detailed conclusions (64). The macaques become viremic soon after SARS-CoV-2 challenge with 3 different virus doses, with viral loads in BAL fluid in the range of 5.3 to 9.0 (median 6.6) log RNA copies/g. There was a moderate relationship between challenge dose and viral loads. In contrast to humans, the macaques largely cleared the infection over days 10 to 28. Anti-S protein endpoint titers were ∼1,000 on day 35, with NAb ID_50_ values of ∼100 in both PV and RV assays. On day 35, the 9 animals were rechallenged with same doses they received in the primary challenge and remained minimally infected or uninfected as judged by viremia and other assessments. However, anti-S protein and NAb anamnestic responses were triggered rapidly, which is a sign that the animals did become reinfected. No correlates of protection could be identified. Hence, at least in the short term (5 weeks) and in an animal model where disease is minimal (see below), SARS-CoV-2 infection is associated with the development of nonsterilizing immunity that reduces viral loads. It is difficult to extrapolate from small-scale animal studies to human SARS-CoV-2 exposure, particularly when the time-dependent decay of immune responses is considered. What would happen if the second challenge were delayed for several months?

Useful information can be derived from studies of common-cold coronavirus infections in humans. Serum IgG and IgA antibodies and NAbs increased by ∼10-fold within 3 weeks of experimental infection with coronavirus 229E, declined markedly over the next 9 weeks, and were at or near baseline levels after one year (65, 66). In a more recent report, 10 subjects were monitored over a 35-year period (1985 to 2020) for their antibody responses (measured using an N-protein fragment) to four different seasonal common-cold coronaviruses (67). Protective immunity waned over time, with substantial reductions in anti-N antibody titers by 6 months postinfection. By 12 months after an initial infection, reinfections were frequent, implying that immunity was not sustained. The probability of infection by month of the year was also assessed, showing that there is a steady decline from May until September (the summer months in this Dutch Northern Hemisphere study) before a steady increase during the winter months (67).

Two experiments were conducted in which humans infected with common-cold coronaviruses were rechallenged several months later (reviewed in reference 15). Six individuals exposed to one strain of the 229E coronavirus became infected, but none was reinfected when challenged with the same strain one year later. However, in a similar experiment, 5 of 8 initially infected volunteers were susceptible to a heterologous strain when rechallenged 8 to 14 months later (66). In a later study, when 15 volunteers were initially exposed to the 229E coronavirus, 10 of them became infected and 8 developed colds. Serum IgG levels were ∼3-fold higher in the uninfected group on the day of challenge. Antibody levels had returned to near baseline values when the same volunteers were rechallenged one year later. All 5 of the originally uninfected volunteers became infected after the second exposure, which was also the case for 6 of the 9 people who had been infected one year earlier (65). The small scale of these studies precludes drawing any strong conclusions about protective immunity other than that it can persist for at least one year, does not occur in all subjects, and may be antibody mediated. These long-ago experiments did not identify antibody titers that protect against common-cold coronaviruses. It was not possible to infer anything about protective titers in a more recent study of coronavirus immunity, as only antibodies to the N-protein were measured (67). In any case, extrapolating from common-cold coronaviruses to SARS-CoV-2 would be difficult, at best, because of the influence of various differences such as neutralization sensitivity and transmission efficiency.

Human antibodies induced in response to infection by common-cold coronaviruses bind only minimally to the SARS-CoV-2 S-protein (15, 68). It seems unlikely that low-level cross-reactivity would affect susceptibility to infection by SARS-CoV-2 or the subsequent COVID-19 disease course, either beneficially or adversely. However, ∼40% to 60% of a cohort of unexposed people had SARS-CoV-2 cross-reactive CD4^+^ T-cells, suggesting that there is the potential for T-cell cross-recognition between common-cold coronaviruses and their more pathogenic cousin (24). In another cohort, 34% of healthy seronegative donors had CD4^+^ T-cell responses to SARS-CoV-2 (compared to 83% of COVID-19 cases), which was attributed to cross-reactivity from responses to common-cold coronavirus infections. The cross-reactive epitopes are most likely in the relatively conserved S2 domain of the S-protein (69). Whether such cross-reactivity might be associated with protection from infection needs to be investigated in larger-scale studies.

Taken together, long-term studies indicate that antibody responses to common-cold and pathogenic coronaviruses are not very long lasting (17, 52, 57, 60, 62, 67, 70). If SARS-CoV-2 behaves similarly, there are significant implications for how long infected individuals resist reinfection, the maintenance of herd immunity in a population, and the frequency with which vaccine booster immunizations may need to be given (see Fig. 3).

## NEUTRALIZING MONOCLONAL ANTIBODIES TO THE SARS-COV-2 S-PROTEIN

Both neutralization-relevant and -irrelevant epitopes are present on S-proteins. As noted above, antibodies to the RBD were detected in the majority of COVID-19 patients and are sometimes strongly neutralizing (17). Intensive efforts to isolate and characterize neutralizing monoclonal antibodies (nMAbs) from COVID-19 cases or experimentally immunized animals are now ongoing, both to better understand the nature of the antibody response in the context of vaccine development and to produce reagents for passive immunotherapy or prevention. As with vaccines (see below), the use of different assays in different laboratories affects, but probably does not preclude, direct comparisons of reported nMAb potencies (50% inhibitory concentration [IC_50_] values).

In an early study on a Chinese cohort, 206 RBD-specific IgG memory B cells were isolated from eight patients (53). The resulting MAbs represented heavy- and light-chain families apparently at random. One patient had coexisting germ line and matured S-reactive clones, with both categories having neutralization activity. Overall, the degree of somatic mutation (SM) was low and did not correlate with affinity. The nMAbs that most strongly competed with angiotensin-converting enzyme 2 (ACE2) binding neutralized most potently (in one case, with an IC_50_ of 30 ng/ml), but the overall correlation was weak, as was the relationship between affinity (dissociation constant [*K_d_*] from the nanomolar range upwards) and competitive capacity. Moreover, some of the nMAbs with the highest affinity for the RBD did not block ACE2 binding (53).

Much more potent nMAbs have now been isolated from a Dutch COVID-19 cohort (25). Blood was drawn from three infected people at about 4 weeks post symptoms, two (COSCA-1 and -2) being mild “at-home” cases, while the third (COSCA-3) had severe disease requiring intensive care. Anti-S protein endpoint titers in the three patients were 13,600, 6,100, and 48,100, respectively, with ID_50_ NAb titers in a PV assay of 383, 626, and 7,645, respectively. Of note is that the highest anti-S protein and NAb titers were in the patient with the most severe disease (see above). S-proteins were used to isolate B cells, leading to 409 paired heavy-chain (HC) and light-chain (LC) clones (137, 165, and 107, respectively, from the above three individuals). SM levels were very low at 1% to 2%, implying that these antibodies have sequences very close to those of the human germ line. All of the HC/LC pairs were expressed in 293F cells, leading to 84 MAbs that were derived mostly from the COSCA 1- and -2 patients. Among them, 32 bound to the RBD, 33 recognized epitopes elsewhere on the soluble S-protein, and several others reacted strongly with S-protein expressed on the cell surface but not with the soluble version. Only 19 of the 84 S-protein MAbs had neutralizing activity in a PV assay, of which 14 were against the RBD. Nine MAbs neutralized at <100 ng/ml, the rest with lower potencies. The RBD-targeting nMAbs COVA1-18 and COVA2-15 were the most potent, with IC_50_s of 8 ng/ml. The best nMAbs had similar potencies in an RV assay. In general, but with exceptions, the RBD-directed nMAbs blocked ACE2 binding strongly. A small number of MAbs modestly (<2-fold) increased infectivity in a concentration-dependent manner, although the sera from the three donors lacked any antibody-dependent enhancement (ADE) activity. The epitopes of the most potent nMAbs were studied in substantial detail using a variety of techniques, with multiple subclusters identified (25).

Comparably potent nMAbs were isolated from a San Diego-based COVID-19 cohort of 22 patients with a range of disease profiles from moderate to severe (27). Anti-S protein titers ranged from very low to >10,000 (note that these are midpoint titers, whereas Brouwer et al. [25] reported endpoints). NAb titers varied from undetectable to >1,000, with excellent concordance between PV and RV assays and a strong correlation between NAb titers and anti-RBD and anti-S protein ELISA titers. Three donors, CC6, CC12, and CC25, were selected for B-cell sorting, which yielded 2,045 IgG antibodies. Only a small proportion of antibodies to the S-protein can neutralize, as many nonneutralizing MAbs were identified. In total, 19 different NAb lineages were identified, and SM was again very low at 1% to 2%. The epitopes for the best 27 MAbs were studied in detail, leading to the identification of three different epitope clusters on the RBD and three more elsewhere on the S-protein. Neutralization titers were highly variable. The most potent nMAbs recognized an epitope designated RBD-A, with the three best having IC_50_s of ∼10 ng/ml in the PV neutralization assay and similar activities against RV. Many of the less-potent nMAbs were also less effective, with maximum neutralization well below 100%, even for some to the RBD-A site. This observation needs to be better understood. The Syrian hamster challenge model was then used to test two of the nMAbs: CC12.1 to the RBD-A cluster and CC12.23 against the S-B site, with NAb IC_50_ titers of 19 ng/ml and 22 μg/ml, respectively (see below).

A New York-area cohort involving 68 convalescing COVID-19 patients also yielded highly potent nMAbs (26). Samples were collected on average 30 days after the onset of symptoms. Plasma anti-S protein and anti-RBD IgG and IgM measurements varied widely, with a strong correlation between the magnitude of the antibody responses and the duration of symptoms. Midpoint NAb titers in a PV assay ranged from 5 to >5,000 (only 2 serum samples reached that high level), with a geometric mean titer of 212 for the cohort. Most plasma samples from patients who recovered from modest disease had only low to modest neutralization activity, with anti-S protein and anti-RBD titers again strongly correlating with NAb titer. An RBD bait was used to isolate B cells from 6 donors, including the two with the highest plasma neutralization titers, leading to 534 HC/LC pairs, of which 34 were then expressed. The very strong sequence similarities among antibodies from different donors has useful implications for the response to S-protein vaccines on a population basis. Of the 34 MAbs, 32 bound to two different subclusters on the RBD, with an average 50% effective concentration (EC_50_) of 6.6 ng/ml, while 20 of them neutralized SARS-CoV-2, with IC_50_s in the range 4.4 to 709 ng/ml. Of note is that some potent nMAbs emerged from donors whose plasma was only weakly or modestly neutralizing. Thus, the serology assays were unable to predict the presence of rare antibody clones with significant neutralization activity (26).

Sixty Chinese convalescent COVID-19 patients were screened to isolate >8,500 B-cell clonotypes that in turn yielded 14 potent nMAbs, all of them against the RBD (74). None of 72 MAbs against other S1 and S2 epitopes was neutralizing. The best nMAb, BD-368-2, had an IC_50_ of 1.2 ng/ml in a PV assay and 15 ng/ml when tested against an infectious virus. At 9.3%, it was more highly mutated from the germ line sequence than the others described above. BD-368-2 inhibits ACE2 binding, as expected, and its cryo-electron microscopy (cryo-EM) structure has been solved as an S-protein complex. Its performance in a passive transfer experiment in transgenic mice is summarized below.

A large set of nMAbs against the RBD emerged from DNA plus S-protein-immunized mice and SARS-CoV-2-infected humans. The most potent among them neutralized a SARS-CoV-2 vesicular stomatitis virus (VSV)-PV with IC_50_ values in the range 7.2 to 99 pM (∼1.1 to 15 ng/ml). Although the nMAbs did not act synergistically in neutralization assays, the best among them will be tested clinically in combinations so as to reduce the potential for the emergence of escape mutants (75).

All five of the above-described studies led to the identification of nMAbs that neutralize SARS-CoV-2 with IC_50_ values in the 1 to 10 ng/ml range via interactions with the RBD (25–27, 74, 75). Apparently less-potent nMAbs emerged from three other efforts. One involved a single patient in the Seattle area from whom 44 MAbs were isolated using an S-protein-based bait at 21 days postinfection (28). The anti-S protein endpoint titer in the donor plasma was ∼10,000 and the NAb ID_50_ in a PV assay was ∼3,000. The MAb sequences were, again, close to those of the germ line. Only 3 of the 44 MAbs bound the RBD, the remainder recognizing epitopes elsewhere on the S-protein. Two of the MAbs had neutralization activity: CV30 had an IC_50_ of 30 ng/ml, bound the RBD, and blocked its interaction with ACE2, while CV1 recognized an epitope outside the RBD but neutralized only partially and with an IC_50_ of 15 μg/ml (28). Another MAb isolation program was based on samples taken at 35 to 50 days post symptoms from four individuals who were infected in China very early in the COVID-19 pandemic and then traveled to the United States (31). NAbs were detected in sera from patients 3 and 4 using an RV assay, with ID_50_ values of ∼100. B cells from these two patients yielded 386 recombinant MAbs that were grouped into 5 categories based on antigen recognition patterns. Most of those with neutralization activity mapped to the RBD, the most potent (MAb 30) having an IC_50_ of ∼300 ng/ml (31). An RBD bait was used to isolate 17 paired B cell clones from a single COVID-19 patient in China (29). Those clones yielded four MAbs that were RBD-reactive in biolayer interferometry (BLI) assays. Their neutralization IC_50_ values in an RV assay ranged from 0.177 to 1.375 μg/ml. Two of these nMAbs, B38 and H4 against different epitopes, were tested in a virus challenge experiment using transgenic mice (see below).

Taken together, the nMAb isolation projects show that highly potent antibodies against the RBD are induced in multiple COVID-19 patients at different stages of disease, including those with only mild symptoms. The dominance of the RBD as a neutralization epitope(s) is consistent with its relatively limited shielding by glycans that substantially occlude the rest of the S-protein’s surface (76, 77). The similarity of the RBD epitopes for these nMAbs and their very limited maturation from germ line sequences are encouraging indicators that potent polyclonal NAbs will be triggered by S-protein or RBD vaccines (25–29, 31, 53). While the most potent nMAbs to the RBD block interactions with the ACE2 receptor, some do not (25–27, 29, 75). One early example is nMAb S309 that was isolated from memory B cells of a patient who had recovered from SARS-CoV-1 infection in 2003, which neutralizes SARS-CoV-1 and -2 with similar potencies (IC_50_ ∼100 ng/ml) by ligating the RBD but does not interfere with ACE2 binding and was ineffective as a Fab (78). Of note is that the conserved S309 epitope involves glycans. Thus, an S-protein vaccine candidate may need to have an appropriate glycan profile to be able to induce this particularly broad NAb specificity. The 47D11 nMAb binds the S1-B domain of the S-protein and neutralizes by an unidentified mechanism that also does not involve competition with ACE2 binding. It was isolated from transgenic mice immunized with a series of CoV S-proteins and neutralizes both SARS-CoV-1 and -2 with EC_50_s in the 0.1 to 1 μg/ml range (79). Other SARS-CoV-1 and -2 cross-reactive nMAbs are also known, but they are not very potent (80, 81).

Other immunogenic epitopes on the S-protein are targeted by a substantial proportion of nonneutralizing MAbs (25–27, 43, 53). The presence of both NAbs and non-NAbs in plasma shows that anti-S protein ELISAs (which detect both categories) are not a perfect surrogate for virus neutralization assays (which detect only NAbs), despite the frequently seen correlations between these two variables. Whether non-NAbs might contribute to ADE and related adverse events is discussed further below.

## IMMUNOGENICITY OF SARS-COV-2 VACCINE CANDIDATES

How good are the leading vaccine candidates at inducing antibodies to S-proteins? What binding antibody and NAb titers can be expected? Here, we review how well various SARS-CoV-1, MERS-CoV, and SARS-CoV-2 S-protein-based vaccines elicit antibodies, including NAbs, in animals and humans (for reviews, see references 1, 8, 15, and 17). As noted above, the outcomes of immunogenicity studies are generally evaluated in different ways. Not only do the assays themselves vary (e.g., whether NAbs are quantified using PVs or RVs), but the resulting data are also presented in a range of formats (e.g., endpoint, midpoint, or unspecified titers or as the extent of neutralization at a fixed serum dilution). Similarly, ELISA data are reported in different ways, including midpoint or endpoint titers, area under the curve (AUC) plots, or signals at a fixed dilution. The lack of standardization can make it very difficult to cross-compare the outcomes of different experiments. However, by using only ELISA endpoint and NAb midpoint (ID_50_) titers, we can compare the magnitudes of antibody responses induced by different vaccines and by SARS-CoV-2 infection (Fig. 2). Inspecting other papers where antibody responses are recorded in other formats reinforces what is shown (see below and the legend to Fig. 2). The generally stronger responses to recombinant protein and killed virus vaccines, compared to those for other concepts, are clearly visible.

A major obstacle to the development of an effective HIV-1 vaccine or a broadly active influenza virus vaccine is sequence diversity in their spike glycoproteins that affects NAb epitopes (82). Variation in the SARS-CoV-2 S-protein sequence has been reported but on a much smaller scale and without, to date, significant implications for the development of an effective NAb response (83–85). Clearly, this is an area for intensive monitoring, on a global scale, but the relatively static nature of the antibody targets on coronaviruses is a welcome aspect of this particular vaccine challenge. Conversely, the SARS-CoV-2 S-protein is highly glycosylated (Fig. 1B). Its glycan content approaches the exceptional density seen on its HIV-1 counterpart and far exceeds what is seen on the influenza virus hemagglutinin (HA) protein (76, 77, 82, 86). The ability of glycans to both shield NAb epitopes and shift their positions under selection pressures should not be ignored. Nonetheless, the hope is that eliciting NAbs against the SARS-CoV-2 S-protein may be fairly straightforward compared with that for, e.g., HIV-1 Env, whose key epitopes are more complex, often more shielded, and much harder to present properly to the immune system (82, 86). The use of the smaller and less glycosylated RBD as a NAb-inducing immunogen could be particularly beneficial in this regard (76, 87).

Awareness of how the same vaccine technologies perform in other viral settings can also provide some insights. Most of the larger industry-based vaccine programs announced to date fall into one of three general categories: nucleic acid (mRNA or DNA) plasmids, replicating virus vectors (adenovirus or vaccinia virus), and recombinant S-proteins or the RBD (8, 14–18). There are also well-advanced killed virus vaccine programs (88, 89). Multiple other technologies are being evaluated by individual research groups, but at a slower pace and with far fewer resources applied (https://www.who.int/who-documents-detail/draft-landscape-of-covid-19-candidate-vaccines). Passive immunization with nMAbs is an alternative approach to protection that is beyond the scope of this article (14–18). However, as for early passive transfer experiments in animals (see below), human studies with COVID-19 plasma or nMAbs may reveal useful information on protective antibody titers.

A major driving force behind the more-prominent programs appears to be the speed at which a vaccine product can be manufactured *en masse*, using existing production facilities (19). Thus, political pressures and societal needs are creating unusually aggressive timelines for the delivery of a product that can be used widely in humans. There are even indications of competition among nation states rather than the more productive cooperative process (19). The relative simplicity with which nucleic acid vaccines can be designed and manufactured has given this technology a substantial head-start in the race to the clinic and beyond. Similarly, viral vector vaccines, notably the adenovirus candidates, were relatively straightforward to repurpose from existing candidates (e.g., for MERS-CoV or HIV-1); these vaccines can be produced in very large amounts in already available facilities. Conversely, producing and purifying recombinant proteins in the doses needed to immunize large populations is likely to be slower and more challenging, and few facilities outside China produce killed virus vaccines in bulk nowadays.

General experience, combined with emerging data, suggests that the most rapidly produced vaccines (i.e., nucleic acids and virus vectors) may also be the least capable of eliciting high titers of antibodies and NAbs to the S-protein (Fig. 2). That is not to say that these vaccine designs will necessarily fail, as it is possible that they will be sufficiently immunogenic to meet the goal of protecting against SARS-CoV-2. Their prospects will be critically dependent on the NAb titer (efficiency) and maximum extent of neutralization (efficacy) needed for protection, how close the immune responses they elicit meet those marks on a population basis, and how long the initial titers are maintained over time (Fig. 3).

In the HIV-1 vaccine arena, adenovirus vaccines induce only weak antibody responses in animals and humans compared to those from recombinant proteins, even when the endpoint is only nonneutralizing antibodies (non-NAbs) (82, 90). Indeed, an adenovirus-based vaccine from Janssen that is now in phase 2b trials for HIV-1 prevention includes a recombinant spike-derived protein boost component that is specifically intended to increase non-NAb titers (it is unable to elicit NAbs in a meaningful way) (90, 91). This adenovirus vector seems conceptually similar to the one that is the basis of the same company’s SARS-CoV-2 vaccine program. A different adenovirus vector (ChAdOx1) that is also part of the Warp Speed programs was only weakly immunogenic in humans when its counterpart was used to deliver the MERS-CoV S-protein. Thus, anti-S protein endpoint titers in the highest dose group were only ∼1,500, while NAb titers varied from undetectable to ∼20 in an intravenous (i.v.) IV assay (92). In a study of a MERS-CoV modified vaccinia virus Ankara vector, the highest geometric mean NAb titer in an RV assay was ∼100 (93). As summarized above, anti-S protein antibody and NAb titers in COVID-19 patients can be orders of magnitude greater than has been achieved in these virus vector immunization studies.

A different adenovirus vector expressing the full-length SARS-CoV-2 S-protein, CanSino’s Ad5 vaccine candidate, has been tested in a phase I human trial (94). Immunogenicity was vaccine dose dependent. In the highest dose group, the geometric mean endpoint anti-S protein titer on day 28 was ∼600, while the anti-RBD titer was 1,400. It was not explained why anti-RBD titers were greater than anti-S protein titers, which is not usually seen. Geometric mean endpoint NAb titers were 34 and 46 in RV and PV assays, respectively, and were strongly correlated with anti-RBD titers. Considering the uncertainties involved in cross-study comparisons, this vaccine candidate does not seem to be very efficient for inducing antibodies to the S-protein in humans. One factor may have been some interference by preexisting immunity to the Ad5 vector itself (94, 95). This problem affected an Ad5 vector-based vaccine that conferred no protection against HIV-1 acquisition in a large-scale trial (96, 97). A substantial number of mild-to-moderate adverse events were reported in the CanSino SARS-CoV-2 trial (94). To the extent that comparisons are possible, the frequency and nature of these adverse events seem to be worse than was found in the HIV-1 Ad5 vaccine trials (96, 97). If there is such a difference and it reflects a property of the SARS-CoV-2 S-protein, concerns could arise about other vaccines based on this protein.

A substantial unknown is the magnitude and nature of the antibody responses that will be elicited by the mRNA vaccines, as this technology is very new and there is only limited information on the designs, safety, and immunogenicity of the major candidates (9, 98, 99). A press release asserts that the Moderna mRNA vaccine induced antibodies to the SARS-CoV-2 S-protein in humans but contains no data that can placed into an appropriate context (Moderna Inc., Cambridge, MA). An animal study is summarized below (98). Like the viral vector vaccines, DNA vaccines against HIV-1 are often used in the prime-boost mode, in which a recombinant protein is administered to increase the generally weak response to several earlier immunizations with its DNA plasmid-delivered counterpart (82, 90). Inovio’s INO-4800 SARS-CoV-2 DNA vaccine has now been tested in mice and guinea pigs (71). This product, like its MERS-CoV predecessor, is based on the S-protein and was delivered by *in vivo* electroporation, a method that involves applying electric fields to muscle and skin tissue, opening membrane channels to allow uptake of the plasmid. Anti-RBD endpoint titers in the mice were ∼2,000 14 days after a single immunization. After 2 doses on days 0 and 14, the midpoint NAb titers on day 21 in an RV assay were in the range of 50 to 150 in one experiment and 240 to 640 in a PV assay in a second study. In guinea pigs, the endpoint anti-S protein titer 14 days after a single immunization was ∼10,000. After 3 doses on days 0, 14, and 28, the median midpoint NAb titers 1 to 2 weeks later were 570 and >320 in PV and RV assays, respectively. IgG capable of inhibiting the binding of the S-protein to ACE2 was purified from the guinea pig sera. Anti-S protein IgG antibodies were also present in mouse and guinea pig BAL samples at endpoint titers of ∼75 and ∼200, respectively. T-cell response assays on splenocytes were also performed (71).

The Moderna SARS-CoV-2 vaccine mRNA-1273 and an earlier MERS-CoV mRNA vaccine have been evaluated in normal and transgenic mice (98). In the MERS-CoV study, the mRNA was formulated as lipid nanoparticles and given at different doses to transgenic mice. It encoded a stabilized (S2P) S-protein, which was more immunogenic when expressed as a full-length membrane-associated protein than as a soluble S2P-foldon protein. The S2P form was also superior to unmodified S-protein. Geometric-mean NAb titers (ID_50_) in a PV assay were ∼15,000, ∼1,000, and ∼300 in the high-, intermediate-, and low-dose groups. When the mice were challenged with MERS, the two higher dose groups were fully protected (judged by weight and viral load [VL] assays), while the lowest-dose group was partially protected. A protective serum NAb titer in the 300 to 1,000 range can be inferred. The equivalent SARS-CoV-2 S2P mRNA was then given at weeks 0 and 3 to 3 different species of mice, again at 3 different doses. After the second dose, the anti-S protein endpoint titers in the highest-dose group were ∼250,000, 30,000, and 1,000,000 in BALB/cJ, C57BL6/J, and B6C3F1/J mice, respectively, with corresponding geometric-mean NAb ID_50_ titers of 820, 89, and 1,100. Two doses of an adjuvanted S-2P protein gave endpoint anti-S protein titers of ∼1,000,000 and NAb titers in the 160 to 890 range in BALB/cJ mice. A single high dose of mRNA in BALB/cJ mice yielded NAb titers with a geometric mean of 320. Various IgG isotype, cytokine, and cellular immunity studies drew the conclusion that the overall immune response was balanced between Th1 and Th2, which was interpreted as beneficial for avoiding adverse events postinfection. Young adult BALB/cJ mice were then immunized and challenged with mouse-adapted SARS-CoV-2. The higher mRNA doses were fully protective against infection, as judged by VL endpoints in different tissues. The NAb titers in the various protected versus nonprotected groups were not listed, which precluded an assessment of the protective titer. A cross-comparison with earlier dosing experiments in the same mice suggests that a NAb titer of ∼800 is protective, but one of ∼80 is not (98).

The immunogenicity of various doses of a SARS-CoV-2 S-protein-based self-amplifying RNA (saRNA) vaccine, encapsulated in lipid nanoparticles, has been assessed in mice (36). Although RNA based, the principle behind this vaccine is different from that of Moderna’s. After 2 immunizations at weeks 0 and 4, serum anti-S protein antibodies were induced in a dose-dependent manner that exceeded 1 mg/ml in the highest-dose group. Information on titers, the more traditional method of data presentation, was not included, but in the same assay, sera from COVID-19 patients were reported to contain anti-S antibodies that ranged from 10 ng/ml to 100 μg/ml, with a median value of 1 μg/ml. If the quantitation method used is accurate, the implication is that ∼10% of the IgG antibodies in the sera of the highest-dose mice were specific to the S-protein. NAb midpoint titers in a PV assay were also immunogen dose dependent, ranged from 5,000 to 100,000, and were correlated with the anti-S protein antibody responses in the same mice (36).

Another variant of the mRNA vaccine concept involves an RNA replicon expressing the full-length S-protein and formulated as a lipid nanoparticle (100). The immunogen was given once to mice at 3 different doses. The two higher doses induced NAbs (PV assay) at ID_50_ titers of 640 and 230 2 weeks later. Weaker binding antibody responses to the S-protein were induced in older mice rather than younger, which may have a bearing on age-dependent immunogenicity. Pigtailed macaques were then immunized either once (high dose) or twice (lower dose at weeks 0 and 4). The single high dose gave NAb ID_50_ titers in the range of 20 to 50 after 4 weeks, which increased to the 100 to 450 range at week 6. In the two-dose regimen, NAb titers in the two immunized animals were 220 and 360 at week 6, and NAbs were also detected at similar ID_80_ (note, not ID_50_) titers in an RV assay (100).

For all of the above-described RNA-based vaccines, it is unknown whether the results obtained in small animals will be matched in humans. The immunogenicity of mRNA and DNA vaccines is generally far stronger in small animals than in macaques, and more so in humans (101, 102). In humans, 3 doses of an S-protein-expressing MERS-CoV DNA vaccine induced peak anti-S protein endpoint titers ranging from undetectable (<10) to 300,000, with peak endpoint NAb titers of <10 to ∼300 that were mostly undetectable 6 months later (103). An Inovio DNA vaccine, also delivered by *in vivo* electroporation, induced only very weak antibody responses to HIV-1 Env proteins in a recent human trial (104).

Recombinant proteins, delivered with an adjuvant, generally trigger stronger antibody responses than viral or nucleic acid vectors. Antibody endpoint titers induced by SARS-CoV-1 and -2 S-protein vaccines can reach ∼100,000 (62, 105). Multiple doses of adjuvanted proteins are generally needed to elicit high antibody titers in animals and humans. The standard human immunization schedule involves 3 doses at 0, 8, and 24 weeks, with similar protocols as used in animals (106–108). While the third dose could be given sooner, this type of schedule would be problematic for a vaccine aimed at a rapid rollout. However, strong antibody responses were elicited in rabbits after only 2 S-protein doses at weeks 0 and 2, which is encouraging. In this experiment, the animals were immunized twice at 14-day intervals with 50 μg of the complete SARS-CoV-2 S-protein ectodomain (i.e., S1+S2), the S1 or S2 fragments, or the RBD with Emulsigen adjuvant (109). The S1+S2 and S2 proteins were produced in insect cells, the other two in HEK 293 mammalian cells. Endpoint ELISA titers to the various S-proteins (except S2) after the second dose were around 100,000. NAb titers in a PV assay were not presented, but a 1:40 serum dilution conferred 80% to 100% neutralization for all the immunization groups except S2. The highest affinity Abs were directed against the RBD.

A much more complex regimen was used to assess a SARS-CoV-2 RBD-Fc protein. The construct was expressed in mammalian cells, conjugated to keyhole limpet hemocyanin (KLH), and mixed with AS01 adjuvant for an immunogenicity study in rats (16). The eight animals were dosed 7 times, daily over a 1-week period, with ever-increasing amounts of the protein; they received a total dose of 500 μg. After a 30-day period, the immunization regimen was repeated using another 500 μg. NAbs were measured in a PV assay, with the data were presented in a nontraditional format. By comparing the extent of PV infection inhibition with that conferred by an ACE2-Ig construct, the authors concluded that the pooled rat sera contained NAbs that were equivalent to a 100 μg/ml (1 μM) concentration of an inhibitor with an IC_50_ of 1 nM. It is not simple to translate this estimate to studies of other immunogens. Of note is that the anti-RBD sera did not mediate ADE under *in vitro* conditions in which this outcome was seen with Zika virus and rat sera raised against it (16).

Mice were given a different RBD-Fc (mouse) fusion construct on days 0, 8, and 13 (100 μg of protein in Alum/CpG adjuvant and then 50 μg in complete Freund’s adjuvant and finally 50 μg in Titermax). By day 26, serum anti-RBD Abs blocked ACE2 binding at dilutions in the 100 to 10,000 range, while NAb ID_50_ titers in a PV assay were ∼10,000. In a follow-up experiment using a simpler protocol, the mice received 5 μg of an Alum-adjuvanted RBD-His protein on days 1, 10, and 25. Anti-RBD endpoint titers by day 40 were 3,000,000, ACE2-blocking titers were also high, and NAb ID_50_ titers in a PV assay were 13,000. In an assay to monitor ADE, the sera did not enhance virus entry into FcR-expressing cells (110).

In another study, mice were immunized on days 0 and 21 with 25-μg doses of the SARS-CoV-2 RBD in “QuickAntibody adjuvant.” The autologous endpoint anti-RBD titer was ∼75,000 on day 35, while midpoint autologous NAb titers in a PV assay were ∼15,000. Titers this high exceed by orders of magnitude what is seen with, for example, nucleic acid-based vaccines in small animals, to the extent that cross-study comparisons are possible (111).

Presentation of proteins as particulate antigens usually benefits antibody responses (112). MERS-CoV RBD-based nanoparticles (NPs) of the SpyTag/SpyCatcher design were ∼10-fold more immunogenic than their soluble protein counterparts when rabbits were immunized in Adjuplex adjuvant on days 0 and 28. Endpoint titers to S-proteins in the RBD-NP group exceeded 100,000 by day 46, while 90% (ID_90_) NAb titers in a RV assay of ∼5,000 compared favorably to ∼500 for the soluble RBD protein group. After challenge with MERS-CoV, nasal swab viremia was reduced by ∼1,000-fold in the RBD-NP recipients but not in the soluble RBD group, which implies titer-dependent NAb-mediated protection (113).

Recombinant protein vaccines are usually given with an adjuvant to boost their immunogenicity, and although details are scarce, it seems likely this would be the case when SARS-CoV-2 proteins are used in humans. Adjuvants vary in potency, with the one most commonly used in humans (Alum) being considerably less effective than newer but less-well-studied alternatives (114). Attention clearly needs to be placed on this area, so that the best possible adjuvant is used, whichever company produces it. It is encouraging, however, that multiple different adjuvants have supported very strong NAb responses to SARS-CoV-2 (and, earlier, to SARS-CoV-1) RBD immunogens in various animals (8, 16, 87, 110, 111). As adjuvants such as complete Freund’s are known to be quite damaging to the structural integrity of proteins, the key NAb epitopes on RBD proteins may be quite robustly presented. Countering this optimism somewhat is the rapid decay of antibody and NAb responses to the SARS-CoV-1 RBD protein in immunized mice, as noted above (62).

## PRODUCTION OF RECOMBINANT S-PROTEIN AND RBD VACCINE CANDIDATES

The most immunogenic vaccine candidates tested to date are recombinant S- and RBD-proteins (Fig. 2). How easily can such proteins be produced in bulk? General experience suggests that constructing stable lines and producing high-quality recombinant proteins will take well more than one year. The SARS-CoV-2 S-protein is highly glycosylated (76, 77) (Fig. 1B). Experience from the HIV-1 field suggests that making large amounts of glycan-rich proteins can be extremely challenging. When properly folded HIV-1 Env trimers are produced by transient transfection of mammalian cells under academic laboratory conditions, the yields are in the range 1 to 5 mg/liter (108, 115). Soluble SARS-CoV-2 S-proteins can be expressed and purified under similar conditions at generally similar levels. The smaller and less-glycosylated RBD is easier to make than the full-length S-protein or the S1 fragment. Thus, the RBD was produced at 25 to 50 mg per liter in Expi293F cells, a 5- to 10-fold higher yield than the S-protein (116). A much greater yield, ∼200 mg/liter, was achieved for the SARS-CoV-1 RBD in yeast cells (117). Fully purified SARS-CoV-2 RBD and S proteins were produced at 30 and 1 mg/liter, respectively, in insect cells, which, like yeasts, express different glycoforms than mammalian cells (118). Whether the characteristics of the glycans matter for immunogenicity can only be determined in comparative studies. The application of structure-guided design principles improved the yield of the S-protein variant by ∼10-fold compared to that of the wild type, with a 5°C increase in thermal stability; the stabilized HexaPro variant was produced at 32 mg/liter in transiently transfected ExpiCHO cells (119). An additional S-protein polymorphism, D614G, increases protein stability and may also benefit production (85).

Mammalian cell lines are likely to be the substrates for large-scale vaccine production, but even if highly producing lines are cultured in industrial facilities, the amounts of immunogens needed will still be daunting. Mice have been immunized with 5-μg doses of SARS-CoV-2 S-proteins or 10 μg of a SARS-CoV-1 RBD-Fc (62), while rabbits were given 50 μg of SARS-CoV-2 S-protein-based immunogens (109) and macaques and rats received 500- and 1,000-μg doses (16, 105). The protein dose used in humans varies, but HIV-1 envelope glycoproteins are generally given in the 100 to 500 μg range. Even if SARS-CoV-2 S-proteins are given to humans at a relatively low dose of 100 μg, a full course of 3 immunizations would require 300 μg of protein. In other words, around 1 g of S-protein would be needed to immunize 3,000 people and, hence, 1 kg for 3 million. It will be no simple matter to produce these amounts of recombinant proteins rapidly. Gram quantities of properly folded HIV-1 trimers were made for phase I trials (115). Larger amounts of earlier generation HIV-1 gp120 subunits were produced for efficacy trials in a few thousand people, but the process was not simple (120). A MERS-CoV RBD construct has been produced in a stable CHO cell line at a final yield, post purification, of 89 mg/liter (121). A stable CHO cell line can express 50 mg/liter of the S1-Fc protein; it has been estimated that a 3,000-liter bioreactor could produce 3 million doses of a human COVID-19 vaccine of this design every 10 days (16). RBD proteins might be produced more efficiently (87).

## VACCINE CHALLENGE EXPERIMENTS IN MONKEY MODELS

Animal model studies involving vaccine immunization followed by virus challenge can provide useful information on the requirements for human protection but are often difficult to interpret unequivocally. Several such experiments in small animals are summarized above. Our experience with the HIV-1 vaccine field over 30 years tells us that the outcomes of animal experiments tend to be emphasized when they support the development of a particular vaccine candidate but dismissed as of minimal relevance when they do not. We see few grounds to believe that this aspect of human nature will be any different for SARS-CoV-2 vaccines. The extensive SARS-CoV-1 and MERS-CoV animal model literature has been thoroughly reviewed (122–124). What it teaches us about vaccine-mediated adverse events is a topic that we address separately below. A comprehensive review of SARS-CoV-2 infection and pathogenesis models is now available (125).

A common finding in HIV-1 animal model research is that it is easier to protect against a virus that replicates inefficiently in the host and that does not cause severe disease than against a more lethal challenge. This scenario may apply also to SARS-CoV-2 animal models (40, 125–127). Thus, the more limited replication of this virus in monkeys may make these animals easier to protect than humans. When 8 rhesus macaques were infected with SARS-CoV-2, they all became sick with signs of lung pathology. Three were killed on day 3 for postmortem analyses, the other 4 postrecovery. However, all 4 living animals recovered between 9 and 17 days postinfection and none died (128). Anti-S-protein endpoint titers were in the range of 1,500 to 3,000, which is at the lowest end of what is seen in human COVID-19 cases and is associated with mild disease (see above). NAb endpoint titers varied from 5 to 60 and were also low compared to that in humans (128). Although the study was too small for a definitive comparison with human COVID-19 disease cohorts, one interpretation is that the rhesus macaque model may best reflect what happens in humans with mild-to-moderate disease and who do not require hospitalization. The African Green Monkey could be a superior model, as SARS-CoV-2 replicates to quite high titers in this species and causes substantial disease as measured by various criteria, including lung pathology (129). The animals did seroconvert rapidly, although as the antibody assays were based on whole virus lysates, titer comparisons to other species are problematic. Earlier studies on SARS-CoV-1 infection also showed that African Green Monkeys were more susceptible to disease than their macaque counterparts (130). In contrast, cynomolgus macaques are less affected than rhesus by SARS-CoV-2 (37).

Four high-profile studies of candidate SARS-CoV-2 vaccine candidates in the macaque challenge model have now been published (35, 88, 89, 131). Superficially, the outcomes were similar; the animals were reportedly protected from disease, although not from infection. There are, however, substantial differences among them. Data from these studies are included in Fig. 2.

The first paper to appear was based on a killed virus vaccine (Sinovac) in an Alum adjuvant (88). When tested initially in mice and rats, anti-S protein endpoint titers exceeded 100,000 and approached 1,000,000 by the end of the immunization schedule. These titers are at the high end of the range measured in COVID-19 cases; the authors recorded a titer of ∼30,000 for one such human serum sample in the same assay. The peak midpoint NAb titers, measured in a RV assay, in these rodents were ∼1,000, far higher than the value of 30 for a human COVID-19 serum sample under the same conditions. The 4 macaques given different doses of the same vaccine responded with peak anti-S protein endpoint titers of 13,000 and NAb titers of ∼50. There was an immunodominant response to the RBD over that for other components of the killed virus vaccine, implying that it might behave in a broadly similar way to an S-protein or RBD subunit vaccine. All 4 macaques became infected after SARS-CoV-2 challenge, but the severity of the (normally mild) disease was reduced compared to that in control animals. Viral loads in throat swabs were also lower in the vaccinated animals than in controls, particularly in the highest-dose group, and continued to decline during the period 3 to 7 days postchallenge when they remained constant in the control animals. No adverse events were reported, either before challenge or after infection (88).

A later report on another killed virus vaccine involved the Sinopharm BBIBP-CorV product (89). Three different doses (2, 4, and 8 μg) in Alum adjuvant were given, once, to BALB/c mice. The peak NAb IC_50_ titer (RV assay) in the higher-dose groups on day 21 was 1,024. Similar titers were induced by a 2-dose regimen, given on days 0 and 7, while 3 immunizations on days 0, 7, and 14 led to NAb titers of ∼4,000. Various 1- and 3-dose regimens were then tested in other species. The higher-dose groups gave the following NAb titers in the 3-dose regimen: cynomolgus monkeys, ∼250; rabbits, ∼400; guinea pigs, ∼400; rats, ∼500; mice, ∼3,000. Safety studies in rats, guinea pigs, and cynomolgus macaques, assessed in various ways at different doses and times, found nothing notable. In the macaque challenge study, the animals (4 per group) were given either 2 μg or 8 μg of the vaccine on days 0 and 14, with median NAb titers of 215 and 256, respectively, at the time of SARS-CoV-2 challenge on day 24. There were no changes in body temperature in the vaccine or placebo groups over the next 7 days, which is indicative of the mild disease course in these animals. Viral loads in throat and anal swabs and postmortem lung tissues were lower by several orders of magnitude (depending on the time point and sample location) in the two vaccine groups than for the placebo, particularly in the higher-dose group. Lung pathology was also reduced/eliminated in the vaccine groups. It is possible but not unequivocally demonstrated that the higher-dosed animals were completely protected from infection (89).

Another macaque study involved the “Oxford vaccine,” a chimpanzee adenovirus construct (ChAdOx1 nCoV-19) that has attracted considerable media attention worldwide. The recombinant virus vector expresses the SARS-CoV-2 S-protein (131). This vaccine induced anti-S endpoint titers of 100 to 1,000 in BALB/c mice and around 1,000 in CD1 mice, which are very low responses at the bottom end of the range seen in COVID-19 human cases. NAb endpoint titers (i.e., not the more usually reported and much lower midpoints) in an RV assay were ∼40 for the BALB/c mice but were undetectable for 2 of the 5 animals; in the CD1 mice, the median titer was 80. In the macaque study, the peak endpoint anti-S protein titers were ∼1,000, which is similar to the peak titer of ∼1,500 seen when the same group’s MERS-CoV adenovirus vector vaccine was tested at its highest dose in humans (92, 131). The median NAb endpoint titer measured in the macaques was ∼40. Taken together, in both mice and macaques, the antibody responses to this live recombinant virus vector seem very weak, which is consistent with how adenovirus-based HIV-1 vaccines perform in macaques and humans unless a protein boost is given (90, 91). All 6 of the ChAdOx1-vaccinated macaques became infected after SARS-CoV-2 challenge, although with fewer symptoms, including reduced lung damage compared to that of the control group. Significant viral load reductions in various tissues were also reported. No adverse events were found, before or after infection, that could be vaccine attributed (131).

DNA vaccines expressing 6 different SARS-CoV-2 S-protein variants, including the full-length S-protein and the RBD, were tested in rhesus macaques (35). The DNA plasmids, without adjuvant, were given intramuscularly at weeks 0 and 3, and the animals were challenged with SARS-CoV-2 at week 6. Median endpoint anti-S protein titers at week 5 varied moderately with the immunogen but were ∼100 for the S-protein and RBD immunogen groups. These titers are ∼10-fold and ∼150-fold lower than recorded in the ChAdOx1 and killed virus studies in the same species, respectively. Midpoint NAb titers induced by the DNA vaccines at week 5 also varied by immunogen, with median values of ∼100 to 200 in a PV assay and ∼20 to 30 when infectious virus was used. The PV NAb assay titers for sera from human COVID-19 cases ranged from ∼20 to 200 in the same assay. The NAb titers in the RV assay seem comparable to those induced by the ChAdOx1 and killed virus immunogens, assuming the different tests have similar sensitivities. When the animals were challenged, all of them became infected as judged by anamnestic antibody responses, although 8 of the 25 DNA vaccine recipients were RNA negative in lung and nasal samples. Viral loads in the other 17 animals were 3 to 4 logs lower than in the 10 control animals. NAb titers were significantly higher in the 8 nonviremic macaques than in the 17 in which viremia was quantified, suggesting that NAbs were a correlate of protection. As in the other three experiments, no adverse events were identified (35).

Few if any of the animals in the above-described macaque experiments were completely protected from infection, although in each case, there was a reduction in the severity of the already mild disease this virus causes in macaques. Viral loads in nasal swabs were, however, comparable between the vaccine and control groups. This observation caused questions to be raised about the efficacy of the ChAdOx1 vaccine (132). However, interpretation of viral load data is complicated by the likely sustained presence of challenge virus RNA in some sites, particularly those accessible by nasal swabs (64). What is particularly surprising is that the similar outcomes were associated with substantial (in some cases >100-fold) differences in antibody titers to the S-protein, with the killed virus vaccines being the strongest immunogens (Fig. 2). Are the antibody responses induced by the ChAdOx1 and DNA vaccines solely responsible for any protection that was conferred? Perhaps cellular immune responses or some other unmeasured factor, such as mucosal IgA, were contributory (Table 1). On a more technical level, it is not clear why very low anti-S protein titers are associated with significant NAb titers in the DNA vaccine experiment but not in the ChAdOx1 study (35, 131). Nonetheless, it can reasonably be concluded that the ChAdOx1 vaccine, whether for MERS-CoV or SARS-CoV-2, is not a strong inducer of antibody responses to the S-protein in macaques, which also seems true of the DNA plasmids (Fig. 2). modified vaccinia Ankara (MVA) vector systems are likely to behave similarly to the ChAdOx1 and DNA immunogens, based on the weak anti-S protein response to their encoded MERS-CoV S-protein in humans (93). These inferences are similar to what has been seen in studies of other vaccines, such as HIV-1 Env, where only protein-based immunogens induce very strong antibody titers (82).

## WHAT IS A PROTECTIVE ANTIBODY TITER FOR A SARS-COV-2 VACCINE AND HOW LONG MIGHT IT PERSIST?

Poorly understood genetic variables affect how different people respond to the same immunogen, which is a key point when vaccinating large populations. A vaccine is useful if the majority of the recipients develop an antibody response that exceeds the protection threshold and, preferably, for a period measured in years not weeks (Fig. 3). Typically, antibody titers vary by well more than 100-fold among people given HIV-1 or influenza virus protein vaccines (82, 133, 134). Antibody responses, neutralizing or not, to SARS-CoV-1 and -2 and MERS-CoV S-protein-based vaccines are similarly variable in animals and humans (35, 62, 88, 92, 93, 103). As an extreme example of how antibody responses can vary across a human study cohort, peak anti-S protein antibody titers induced by a MERS-CoV DNA vaccine ranged from 3 to 300,000, and in many volunteers, no antibodies were detectable at most time points (103). Thus, a key parameter is where a protective titer lies compared to the range of responses induced by the various vaccine candidates (Fig. 3). Do only the strongest responders or most of them exceed a protective threshold? For how long?

We do not know what magnitude of a vaccine-elicited antibody response could protect humans from SARS-CoV-2 infection and/or severe disease. In the small-scale rhesus macaque SARS-CoV-2 rechallenge experiment referred to above, the two apparently protected macaques had NAb midpoint titers of 8 and 16 in a RV assay on the day of their second challenge. Although anti-S antibodies were measured, no titer data were reported (63). No correlate of protection could be identified in either this study or the somewhat larger one of a similar design (63, 64). Information on possibly protective NAb titers may emerge in the coming months from passive immunotherapy studies in which plasma samples from recovered COVID-19 patients are infused into those with active infection (44, 135–137). The NAb and binding antibody titers infused could be compared with the observed clinical outcomes, although any relationship to vaccine-mediated protection will be imprecise.

The ACE2 proteins of multiple animals have been sequenced and their abilities to bind the SARS-CoV-2 S-protein modeled (138). The modeling includes species possibly relevant to cross-species transmission (bats, pangolins, civets, and raccoons), domestic pets (cats, dogs, and tigers), farm animals (cows and sheep), and possible infection models (hamsters, mice, guinea pigs, and ferrets). Future model systems may emerge from this kind of analysis. In addition to the macaque experiments reviewed above, various small animal models have already been used in SARS-CoV-2 challenge experiments. When Syrian golden hamsters were exposed nasally to SARS-CoV-2, virus was detected in the lungs by day 2, but the animals cleared the infection by day 7 and fully recovered (139). NAb titers of 1:640 were measured on day 7 using an RV assay. The infection can be transmitted, via shared air, to other hamsters in adjacent cages, which is a useful feature that could also be exploited for vaccine efficacy studies (139). Transgenic human ACE 2 (hACE2) mice can be infected by SARS-CoV-2, leading to modest disease that includes lung damage associated with infiltration of macrophages and lymphocytes (140). Both of these small animal models have been used in nMAb passive transfer and challenge studies summarized below (27, 29).

Several human nMAbs have now been evaluated for passive protection of small animals or rhesus macaques (27, 29, 74, 141). The caveats expressed above about vaccine protection in animal models applies also to passive immunization experiments, which complicates quantitative extrapolations to human protection. Two nMAbs were tested in the Syrian hamster challenge model (27). MAb CC12.1 is to the RBD-A site, with a NAb IC_50_ titer of 19 ng/ml, while CC12.23 recognizes the S-B epitope and is ∼1,000-fold less potent, with an IC_50_ of 22 μg/ml. Five different doses were delivered intraperitoneally to the animals, which then received an intranasal SARS-CoV-2 challenge 12 h later. Animal weight was used as an endpoint to measure disease as were viral load assays on lung tissue postmortem (day 5). The potent CC12.1 nMAb conferred dose-dependent protection from disease. There was a trend toward greater weight loss than with a control MAb in the animals given the lowest doses of CC12.1, which is a potential concern because of the possibility of ADE (or similar) at a subthreshold NAb dose (see below). Pharmacokinetic measurements show that a serum antibody concentration of 22 μg/ml was required for full protection, which corresponds to 1,200 times the neutralization IC_50_ in the PV assay (for 50% protection from disease, the values were 12 μg/ml and 630 times the IC_50_). The much-less-potent CC12.23 nMAb was not protective at any dose, further indicating that protection correlates with dose-dependent neutralization (27).

Single doses of the B38 and H4 nMAbs were administered to hACE2 transgenic mice followed by SARS-CoV-2 challenge 12 h later, with body weight and viral load serving as endpoints (29). B38 (IC_50_, 180 ng/ml) was modestly effective at reducing the weight loss, but the viral loads were significantly suppressed ∼1,000-fold.

The BD-368-2 MAb (IC_50_ of 1.2 ng/ml in a PV assay) was also tested in hACE2 transgenic mice (*n* = 3 per group) both for therapy and prevention (74). Given at 20 mg/kg 24 h prior to SARS-CoV-2 challenge, the MAb blocked infection completely as judged by viral load measurements in the lung, although there was a modest weight loss that might indicate low-level infection. When BD-368-2 was instead given 2 h after challenge, there was a similarly modest weight loss, but the mice did become infected albeit with a 3 to 4 log reduction in lung viral load compared to that in the control. Protective antibody doses could not be inferred from this study, but it does suggest that the window for complete protection may be quite short if nMAbs are used for postexposure prophylaxis.

Anti-RBD MAb CB6, with an IC_50_ value in the range of 20 to 50 ng/ml that depends on the assay used, was evaluated in rhesus macaques after modification of its Fc region to reduce the risk of ADE (141). A single dose of 50 mg/kg intraperitoneally (i.p.) given 1 day prior to challenge conferred substantial protection from infection, as judged by VLs in throat swabs. When the same dose was administered 1 and 3 days postchallenge, the rate of decrease of VL was significantly greater than in the control animals (which naturally clear the virus within 7 days). Lung damage was also lower in the MAb recipients (141).

We noted above that complete protection of Syrian hamsters against SARS-CoV-2 challenge required a serum nMAb concentration equivalent to 1,200 times the neutralization IC_50_ (27). For comparison, a comprehensive meta-analysis of nMAb passive immunization experiments showed that 95% protection of macaques from mucosal simian-human immunodeficiency virus (SHIV) challenge required serum nMAb levels 680-fold greater than the ID_50_ (142). Although there are obvious differences between intranasal SARS-CoV-2 infection of hamsters and rectal SHIV infection of monkeys, the quantitative aspects of passive nMAb protection seem quite similar. When 5 mice were immunized with the SARS-CoV-1 RBD protein and then virus challenged, 4 were apparently completely protected and the fifth partially. The serum ID_50_ NAb titer in the infected mouse at the time of challenge was 57, while the titers in the protected animals ranged from 189 to 505 with a mean value of 390 (62). A broadly similar serum ID_50_ NAb titer of 500 was associated with 90% protection against rectal challenge of macaques after active vaccination with an HIV-1 envelope glycoprotein trimer (107). In a conceptually similar experiment, the same envelope glycoprotein trimers induced protective serum NAb titers of ∼300. However, when the animals were immunized with a viral vector vaccine before boosting with the trimer, durable protection was achieved at substantially low NAb titers (143). A combination prime-boost vaccine incorporating components that induce both cellular immune responses and NAbs (e.g., a viral vector or a nucleic acid plasmid plus a recombinant RBD or S-protein) might be worth exploring.

Almost all attention has been placed on measuring antibody responses to vaccines in serum. However, SARS-CoV-2 levels in blood are very low, both in absolute terms and compared to that in other body fluids such as nasal secretions (49). This virus is, of course, usually transmitted via mucosal surfaces, where IgA antibodies are a substantial source of immunity. Very little is known about the mucosal IgA response in COVID-19 cases or after experimental vaccination, which are gaps that warrant filling. In one report, IgA antibodies to the S-protein were found in nasal swabs, tears, and saliva from a few health care workers who were exposed to SARS-CoV-2 but remained uninfected; in general, the mucosal IgA responses were stronger in younger people than in older ones (50). The possibility exists, therefore, that some virus-exposed people may develop mucosal immunity without becoming systemically infected or seroconverting. In this context, a proposal to focus SARS-CoV-2 vaccine development more on mucosal immune responses is worth considering (144). Passive transfer experiments with mucosally administered IgA antibodies seem worth pursuing. An engineered IgA version of an nMAb, with moderate potency, has been described (80).

Antibody responses to coronavirus infection are not particularly long lasting (see above). Hence, another key unknown is how long any protective response to a SARS-CoV-2 vaccine might last. Active or passive immunization experiments in animals almost always involve virus challenges when the antibody titers are at or near their peak values (Fig. 2 and 3). This scenario would rarely apply to vaccinated humans. The few human studies of MERS-CoV and SARS-CoV vaccines show that anti-S antibody titers decline fairly rapidly (within months) from the peak, although detailed information on the decay rates is not available (92, 93, 103, 131). As noted above, the binding antibody titers to the SARS-CoV-1 RBD protein in mice declined by ∼15,000 over a 9-month period (62). Obtaining data on the medium- and long-term antibody decay rates in SARS-CoV-2 vaccinated humans will be essential. It is possible that exposure to SARS-CoV-2 will trigger rapidly protective recall responses even months to years after the course of vaccination. Alternatively, frequent boosting regimens may need to be used.

In summary, it is not known what benchmark serum antibody and NAb titers must be reached for a SARS-CoV-2 S-protein vaccine to protect humans. The animal challenge experiments reviewed above suggest that a serum NAb ID_50_ titer in the approximate range of 100 to 500 is required for sterilizing immunity (i.e., complete protection from acquisition). If so, this magnitude of response in a human population may be best achieved by a recombinant S-protein or, arguably better, an RBD immunogen (Fig. 2 and 3). It is, conceivably, more feasible to induce B-cell memory responses that might protect from disease but not from acquisition. If the early observations in macaques hold true for humans, protection from disease might be the best that the Warp Speed vaccines can accomplish anyway. It also remains to be seen how long protective immunity might persist, but regular booster immunizations may be necessary (Fig. 3).

## VACCINE-MEDIATED ADVERSE EVENTS

If a vaccine confers protection to almost all of its recipients and has no deleterious effect in the minor proportion of people it fails to protect, there are few grounds for concern. No vaccine is fully protective for a large population, but creating herd immunity against SARS-CoV-2 may require a vaccine efficacy rate of only ∼70% if the basic reproductive number for the infection in a naive population is ∼3 (70, 145). It would take ∼1 million deaths for this degree of herd immunity to be achieved in the United States without a vaccine, and manyfold more for a protective outcome worldwide (70). Such estimates, of course, assume that SARS-CoV-2 infection does confer long-lasting immunity (see above).

Whereas a lack of efficacy is clearly undesirable, a vaccine used on a large scale that increases the risk of acquiring an infection or that exacerbates disease postinfection would be disastrous. A poorly protective vaccine will lead to the infection of many individuals who have already mounted antiviral immune responses. In particular, vaccinating during a pandemic could involve a scenario in which weak and potentially deleterious priming responses are induced in people who then encounter the virus before they receive their boosting immunizations (57). In a recent review of what was observed with several SARS-CoV-1 and MERS-CoV vaccines in virus-challenged animals, 36 research papers were identified that reported adverse outcomes, including but not limited to lung pathologies (123). Other reviews have also listed multiple examples of adverse events in coronavirus vaccine experiments (2, 4–6, 17, 122, 146–148). Severe disease caused by SARS-CoV-1 tends to occur around week 3 after infection, when the viral load in the respiratory tract diminishes as NAb titers rise (149). As summarized above, the inverse correlations for the magnitude of the antibody response are seen in both SARS and COVID-19 cases. Taken together, there are concerns that the antibody responses to SARS-CoV-1 and -2 may not protect against disease but could even contribute to pathogenesis (see above).

One widely discussed area of concern is ADE. For some viruses, such as dengue and West Nile viruses, antibodies can enhance the degree of infection of the standard target cells by ligating proteins on the viral surface while also interacting with Fc receptors (or indirectly with complement receptors) on the cell surface. The outcome is to increase the uptake of viruses into the endosomal compartment, where receptor-mediated membrane fusion leads to productive infection of the cell. ADE can be mediated by non-NAbs or by ineffective NAbs when their occupancy of viral spikes is too low for neutralization. Only antibodies that bind to epitopes exposed on the virion surface can mediate ADE, and it is not clear how they could do so without interfering with infection; one possibility is via binding to nonfunctional S-proteins (78, 150). Whatever the mechanism, a previous infection with an antigenically related virus or a vaccine that induces non-NAbs, or inadequately effective or poorly persistent NAbs, could cause ADE (4, 150). ADE was responsible for the adverse outcomes of some dengue virus vaccine trials (42).

The risk of ADE for SARS-CoV-2 is a topic for serious discussion (2, 4–6, 8, 17, 73, 122, 123, 146–148). However, the evidence for ADE arising in SARS-CoV-1 and -2 and MERS-CoV experimental infections and vaccination studies is ambiguous (reviewed in references 4 and 17). It should be noted that, for these coronaviruses, the mechanism for ADE may differ from what applies to viruses whose standard target cells are of the myeloid lineage and express Fc receptors. In contrast, SARS-CoV-2 primarily infects pulmonary, endothelial, renal, and intestinal parenchymal cells that express ACE2. In these circumstances, Fc receptor-mediated ADE would not only enhance infection of already susceptible cells but also could expand tropism to, e.g., monocytes and macrophages, thereby changing the already complex disease course. There are various examples of this scenario. Strong ADE was observed in studies of feline infectious peritonitis virus (FIPV), a macrophage-tropic coronavirus that triggers systemic vasculitis (151). Immunization of cats with a vaccinia vector expressing the cognate S-protein increased death rates after FIPV challenge (4, 152). Antibodies elicited when rodents were immunized with the SARS-CoV-1 S-protein enabled the virus to now enter human B-cell lymphoma cells *in vitro* in an ACE2-independent FcR-dependent manner, although this did not lead to productive infection (153). A nMAb to the MERS-CoV S-protein neutralized ACE2-mediated entry but could also enhance FcR-dependent entry in model cell lines (154). Serum from SARS-CoV-1-infected patients with S protein-specific antibodies facilitated virus infection of macrophages *in vitro* (72).

In contrast to the above-described examples, ADE was not seen after animals were immunized with the SARS-CoV-1 RBD (62) or with inactivated SARS-CoV-2, vector-expressed S- protein, or recombinant RBD (16, 35, 88, 110). Even with flaviviruses, ADE detected *in vitro* does not always translate into enhanced disease *in vivo* (4). Likewise, multiple passive immunization studies in mice and nonhuman primates have failed to show signs of ADE *in vivo* upon challenge with SARS or MERS coronaviruses (123), although there was a trend toward greater weight loss when a poorly neutralizing MAb was tested in a Syrian hamster SARS-CoV-2 challenge model (27).

A rational approach to avoiding ADE is to minimize the induction of poorly or nonneutralizing antibodies by using the RBD to focus the antibody response on its key NAb epitopes (16, 87, 123, 147). However, all of the leading Warp Speed vaccine candidates involve the full-length S-protein, which expresses both NAb and non-NAb epitopes.

A concept related to ADE has been termed antibody-mediated enhanced respiratory disease (ERD) (2) or vaccine-associated enhanced respiratory disease (VAERD) (6). While ADE may be relevant to this scenario, so might other immunopathological aspects of vaccine-induced immunity. One clinical manifestation of COVID-19 is a dramatic decline in respiratory function, which occurs in some patients around 7 to 14 days after symptoms appear. That timeline mirrors the onset of seroconversion, and there are data suggesting that the formation of immune complexes between antibodies and virions might activate monocytes and macrophages to trigger a cytokine storm (reviewed in references 44 and 17). In principle, vaccine-induced antibodies could have similar pathogenic effects, in some cases via an FcR-dependent mechanism (51). Complement activation by all three pathways has also been implicated in lung pathogenesis (155, 156). Drivers of the complement pathways are mannose-binding lectin (MBL) and related innate factors that recognize carbohydrate structures on viral spike glycoproteins, including SARS-CoV-1 and -2, HIV-1, and Ebola virus (157–159). This pathway has been implicated in the pathogenesis of Ebola virus infection (157).

Vaccination of humans against the pneumovirus respiratory syncytial virus (RSV) and the paramyxovirus morbillivirus that causes measles provides additional concerns about the potential for VAERD in the SARS-CoV-2 context. The exacerbated pathogenesis observed in the 1960s principally involved killed virus vaccines (6, 160–163). Among children receiving an inactivated RSV vaccine, 80% were hospitalized after infection compared to only 5% of the placebo controls (161). What mechanisms were responsible? First, it has been argued that a high ratio of binding antibodies (i.e., non-NAbs) to NAbs yields immune complexes and detrimental complement activation. This mechanism was shown to be relevant in infants vaccinated with formalin-inactivated RSV who then became RSV infected; complement activation was associated with inflammation and airway obstruction (6, 162). A similar pathology was seen in macaques immunized with an inactivated measles virus (163). Second, vaccination can prime for allergic reactions that are triggered after infection with the corresponding virus. The ensuing pathogenesis comprises increased production of interleukin 4 (IL-4), IL-5, and -13, eosinophil recruitment, and impeded cytotoxic T lymphocyte (CTL) responses (i.e., Th2 polarization), leading to pulmonary dysfunction (6, 164).

What might these observations mean for COVID-19 vaccines? Vaccination with inactivated SARS CoV-1 and MERS-CoV and with the SARS-CoV-1 S-protein has also yielded histopathological pulmonary and hepatic manifestations in various animal models (reviewed in references 148 and 123). One study in particular highlights the risks of VAERD. Here, SARS-CoV-1 S-protein-specific antibodies were elicited by immunization of rhesus macaques with a vaccinia vector followed by autologous viral challenge. The outcome was severe acute lung injury, extreme pulmonary accumulation of monocytes and macrophages, and elevated cytokine secretion. The underlying mechanisms were difficult to determine but could involve ADE augmented by additional immunopathology and may be at least partly FcR dependent and involve immune complex formation (51). Based on histopathological analyses of pulmonary parenchyma, however, it is uncertain whether the observations made in macaques apply to SARS-CoV-2-infected humans. Thus, the lung tissues of both postmortem COVID-19 cases and studies of asymptomatic infections showed prominent infiltration by lymphoid cells but not by macrophages or monocytes (reviewed in reference 122). To date, no adverse events of the above-described nature have been reported in SARS-CoV-2 vaccine challenge studies in macaques (see above).

Some pathogenic effects seen in SARS-CoV-1 and MERS-CoV animal vaccinations have been linked to strong Th2 in relation to Th1 responses (5); the former, promoted by adjuvants such as Alum, have been associated with eosinophil accumulation in lungs (4, 5, 147). The eosinophilic histopathology notwithstanding, Th2-polarized responses to SARS-CoV-1 virus-like particle, inactivated virus, and DNA-delivered S-protein vaccines in mice can be partially protective by reducing viral loads postinfection (165). Some adjuvants, such as Toll-like receptor agonists and inulin, have been suggested to shift Th2 responses to Th1 and reduce VAERD (5, 123, 166). Furthermore, the murine IgG subclass profile is linked to Th polarization, which therefore could affect the FcR interactions of the elicited IgG antibodies (5). The main human IgG subclass, IgG1, is not associated with Th polarization, and there are many other species differences that influence viral tropism and virus-immune system interactions. The murine and other small animal models may, therefore, be problematic for understanding SARS-CoV-2 infection of humans and how vaccines perform. The weaker and less sustained replication of SARS-CoV-2 in macaques, compared to that in humans, could limit the development of ERD/VAERD in this species. Despite these limitations, when animal models are used to derive vaccine efficacy data, as much safety data as possible should also be obtained both before and after experimental infection (148).

In normal circumstances, there would be an extensive assessment of the kinds of adverse events noted above to better understand the interplay between SARS-CoV-2 and the human immune system and to minimize the risks to the vaccinated population. The exceptional circumstances of the COVID-19 pandemic are reducing the time that would normally be taken to analyze critical aspects of vaccine development. Will Institutional Review Boards have all the information required to judge the safety of novel vaccines with limited safety data (9)? Will vaccinated humans be placed at serious risk of harm when they encounter SARS-CoV-2? It is unlikely that ERD/VAERD events will be assessed until a sufficient number of infections occur in vaccinated people during efficacy trials, as too few infections may occur at the phase I/II stages. There are now specific recommendations for how immunogenicity trials in animals and safety trials in humans should be conducted and what information should be sought (148).

Altruistic volunteers are willing to be vaccinated and then challenged with SARS-CoV-2, a scenario that raises difficult ethical questions that are being debated now at some length (12, 167–171). The major beneficiaries of a SARS-CoV-2 vaccine will be older people who are at the most serious risk of death from COVID-19. However, most proposals for human challenge studies involve young healthy people. Would their experience after vaccination appropriately mimic what might happen in an older population with preexisting conditions that render them particularly vulnerable to severe COVID-19? These quite complex scenarios will need to be analyzed from multiple perspective by decision makers with qualifications in the relevant areas of science and public health.

## SCENARIOS FOR FAVORABLE AND UNFAVORABLE OUTCOMES

The most favorable outcome, and the one that all vaccine researchers would like to see, is that the first large-scale efficacy trials show that SARS-CoV-2 vaccines confer robust protection that will bring a speedy end to the pandemic. In favor of that scenario is the presence of what seem to be immunodominant neutralization epitopes on the S-protein’s RBD that are well represented in the human germ line. NAbs to these sites may, therefore, be induced quite efficiently by S-protein-based immunogens. The relative lack of S-protein sequence variation is another favorable factor for vaccine success. Protection could arise either by the induction of a serum antibody titer that exceeds the protective threshold (presently unknown, but see Fig. 3) for a meaningful period or if an antibody response is primed that can be rapidly recalled on systemic exposure to SARS-CoV-2. Cellular and/or mucosal immune responses to some vaccine components may also contribute to protection. It is feasible, but by no means certain, that vaccines that can be relatively quickly manufactured in bulk (e.g., mRNA, DNA, and adenovirus vectors) will be sufficiently immunogenic to elicit protective NAb responses in a high proportion of the population.

An undesirable outcome will be if the first vaccines tested are not immunogenic enough to be protective but are not associated with significant adverse events before or after SARS-CoV-2 infection. Recombinant S- or RBD-proteins are markedly more immunogenic than the current Warp Speed mRNA or adenovirus-based vaccines (Fig. 2). Follow-on trials of these proteins, used alone or in combination with the earlier candidates (i.e., prime-boost strategies), could provide the answer. The most substantive concern here is the time lost in the face of a spreading pandemic.

There is, however, a foreseeable outcome that could set back the wider vaccine field for decades. If the first-tested vaccines fail to protect most recipients but prime or trigger an antibody or other immune response that exacerbates COVID-19 disease in people who become infected, there will be a ferocious public backlash against vaccines in general. The Warp Speed COVID-19 vaccine trials are of enormous interest to our society and are receiving constant attention from the press and public. A small but vocal faction that opposes vaccination for irrational reasons would become even more energized by adverse events and, in the politically polarized America of 2020, could receive high-level support. The consequences could be serious harm not just to the prospects for a successful COVID-19 vaccine but also for the uptake of the commonly used vaccines that are essential to the health and wellbeing of our children. The stakes are high. A powerful Opinion piece in the New York Times argues strongly for the need to obtain the most comprehensive data set possible on the potential risks of SARS-CoV-2 vaccines and urges the FDA to not issue an emergency-use approval based solely on immunogenicity data (https://www.nytimes.com/2020/06/08/opinion/trump-coronavirus-vaccine.html). We wholeheartedly agree.

## CONCLUSIONS

A protective vaccine against SARS-CoV-2 is a goal that is achievable but by no means certain. Although SARS-CoV-1 vaccine development gradually petered out once that virus stopped spreading in humans, considerable efforts are thought to have been made in Saudi Arabia over the past 8 years to develop a MERS vaccine to protect commercially valuable camels and horses. No such vaccine has ever emerged. The various SARS-CoV-2 vaccine designs are associated with perceived advantages and drawbacks (Table 1).

For the aggressive timelines of the Warp Speed program to be met, very little can go wrong at any stage of the research and development processes. Few if any large-scale projects proceed smoothly, particularly when there are major and quite fundamental gaps in the underlying science. Moreover, obtaining a rapid endpoint in an efficacy trial requires a high incidence of infection in the area of the trial sites, but infection rates are now declining in many areas of the United States and Europe where leading research institutions are located. Conducting trials in areas of the world where infection rates are still high, or even increasing, would overcome such concerns. Recent media reports suggest that efficacy trials of vaccines from both American and Chinese programs will involve sites in Brazil, a currently high-incidence country. The primary endpoint in the Moderna mRNA vaccine phase 3 trial is prevention of symptomatic COVID-19 disease, while secondary endpoints include prevention of severe disease (hospitalization) and prevention of infection. Quantifying a disease reduction endpoint rather than sterilizing protection from infection could be an additional complication, which is, perhaps, portended by the performance of early vaccine candidates in animal models (see above). That complexity would be exacerbated if effective antiviral drug combinations, including nMAbs, become the immediate standard of care for people with SARS-CoV-2 infection. As noted above, a COVID-19 vaccine is most needed for the more vulnerable populations, which include people who are older (particularly those >70 years) and/or those with preexisting health conditions. Age and perhaps some health concerns may adversely affect the development of immune responses to vaccines (172). Testing a vaccine in predominantly young and healthy volunteers may not predict what happens in their older and sicker counterparts.

If protection against SARS-CoV-2 requires only fairly modest serum antibody titers, then the most easily produced vaccine designs could succeed. But if much higher titers are needed, those vaccines may need to be replaced, or supplemented, by other components that are perhaps produced by another company or in a different country. For example, an American mRNA vaccine may work better if boosted by a Chinese killed virus preparation or a British adenovirus vector when followed by a recombinant protein made within the European Community. Even if an effective vaccine is identified, it may be challenging to manufacture and distribute on the scale needed to immunize a significant fraction of the world’s population (7, 173) (Table 1). An effective vaccine that is too complex to make in bulk, is difficult to formulate, is highly unstable without refrigeration or freezing, is difficult to administer, or that requires too many doses over a prolonged period may represent a Pyrrhic victory for science but not the answer to the problems faced by the societies that science serves. The complexities of developing a vaccine at ultrashort notice are best tackled by the melding of minds irrespective of wherever the bodies are geographically located (173, 174). Will this happen? We hope so, but fear it may not (19).

## ADDENDUM IN PROOF

We recommend consulting reference 175 with respect to the immunogenicity of MERS-CoV full-length S DNA and S1 protein in mice and macaques. We regret being unaware of this paper when we created Fig. 2.
